# Do pollutants and meteorological factors trigger more psychiatry admissions?

**DOI:** 10.1192/j.eurpsy.2025.1190

**Published:** 2025-08-26

**Authors:** E. López Bardón, M. Menéndez Muñoz, A. Serrano García

**Affiliations:** 1 Servicio de Psiquiatría, Complejo Asistencial Universitario de León, León, Spain

## Abstract

**Introduction:**

The high prevalence of mental health disorders makes investigating their etiology a fundamental activity. Factors influencing their physiology include genetic predisposition, substance use, environmental factors, etc. Among these, weather and atmospheric pollutants are two of the least studied. The biologically active agents in the atmosphere interact with living beings, disrupting their homeostasis and leading to alterations in both physical and mental health. This interaction was already noted in the Corpus Hippocraticum in the 4th century BC.

**Objectives:**

The objective is to analyze the impact of various environmental factors and atmospheric pollutants on daily emergency hospital admissions for mental disorders in the healthcare area of the province of León from 2011 to 2022.

**Methods:**

An observational, retrospective, ecological, longitudinal, and time series study is conducted. Admission data is sourced from the coding office at the Complejo Asistencial Universitario de León, meteorological data from the Agencia Estatal de Meteorología, and pollutant data from the Junta de Castilla y León. A combined database in Excel is created for statistical analysis, both descriptive and analytical (Poisson regression), using the SPSS statistical package.

**Results:**

It was observed that hospital admissions for mental disorders are significantly related to sunlight hours, ozone, precipitation, average wind speed, CO, NO2, maximum atmospheric pressure, NO, and PM10.

**Conclusions:**

The findings of this study show that meteorological factors and atmospheric pollutants are related to hospital admissions for mental disorders. Applying this information in psychiatric emergencies could improve forecasting and resource management during periods of higher demand.

**Disclosure of Interest:**

None Declared

